# The Imaging Appearance of *EWSR1::PATZ1* Gene Fusion Central Nervous System Tumors

**DOI:** 10.5334/jbsr.3431

**Published:** 2024-11-20

**Authors:** Cedric Vanmarcke, Lukas Marcelis, Isabelle Vanden Bempt, Raf Sciot, Johannes Devos

**Affiliations:** 1 Department of Radiology, UZ Leuven, Leuven, Belgium; 2 Department of Pathology, UZ Leuven, Leuven, Belgium; 3 Department of Human Genetics, UZ Leuven and KU Leuven, Leuven, Belgium; 4 Department of Pathology, UZ Leuven and KU Leuven, Leuven, Belgium; 5 Department of Imaging & Pathology, Translational MRI, KU Leuven, Leuven, Belgium

**Keywords:** *PATZ1*, *EWSR1*, neuroepithelial tumor, MRI, glioneural tumor

## Abstract

*Ewing Sarcoma Breakpoint Region 1* and *POZ/BTB And AT Hook Containing Zinc Finger 1* (*EWSR1::PATZ1*) gene fusion central nervous system (CNS) tumors are increasingly recognized as a potential distinct entity, with only limited reported cases. The imaging characteristics of these tumors have not been well established. In this study, we provide a detailed radiological description of a case in a 24‑year‑old man and conduct a literature review to identify common imaging features.

A total of seven cases, including our own, were evaluated. Histopathological diagnoses included two ependymomas, an infantile glioblastoma, an astroblastoma, a ganglioglioma, and two gliomas not otherwise specified. Common imaging patterns included avid contrast enhancement, intratumoral cysts, intraventricular location or extension leading to hydrocephalus, and sharp delineation. Additional frequently observed features included calcifications and hemorrhagic foci.

In conclusion, although the histopathological appearance of *EWSR1::PATZ1* gene fusion CNS tumors is diverse, there are consistent imaging features. Recognition of these features can be valuable in the diagnostic process, as radiologists can be the first to suggest the diagnosis.

## Introduction

Historically, the diagnosis of central nervous system (CNS) tumors relied on histological findings and immunohistochemistry. However, since many tumors share morphological features, additional molecular genetic analysis is required. These techniques have redefined the diagnostic landscape, as reflected in the 2021 World Health Organization (WHO) classification of CNS tumors [1, 2].

Molecular diagnostics have identified novel genetic aberrations in CNS tumors. Among those is a fusion of the *Ewing Sarcoma Breakpoint Region 1 (EWSR1)* and *POZ/BTB And AT Hook Containing Zinc Finger 1 (PATZ1)* genes. The *EWSR1::PATZ1* fusion was already known in soft‑tissue sarcomas and was first discovered in a CNS tumor (specifically, in a ganglioglioma) in 2016 [3]. Since then, 21 tumors with this fusion have been described in case reports and molecular profiling studies [2, 4–8], and some authors have suggested *EWSR1::PATZ1* gene fusion tumors may define a new distinct glioneuronal tumor entity [5].

The histopathological appearance of tumors harboring the *EWSR1::PATZ1* fusion is diverse, encompassing entities such as ependymomas, high‑ and low‑grade astrocytomas, glioblastomas, and gangliogliomas. The heterogeneous histopathological and immunohistochemical character‑istics of these tumors pose challenges in determining their cell lineage, whether glial or neuroglial in origin. To reflect this uncertainty, Qaddoumi et al. proposed the term “neuroepithelial tumor with *PATZ1* fusion” [3]. It has not yet been incorporated into the WHO 2021 classification, as there remains uncertainty regarding whether this entity is a distinct tumor type [1]. Notwithstanding their remarkable heterogeneity, certain histopathological features consistently recur within these lesions [4]. The imaging characteristics of this tumor group have not been comprehensively assessed. As *EWSR1::PATZ1* fusion CNS tumors might be a distinct entity, this study aims to investigate the imaging features in documented cases to identify common imaging patterns that may aid radiologists in suggesting a diagnosis.

## Case Report

A 24‑year‑old male presented to our tertiary academic hospital’s emergency department with persistent headaches lasting 1 week, accompanied by intermittent vomiting, kinesiofobia, and phonophobia. His medical history, neurological examination, and laboratory tests were unremarkable.

**Figure 1:**
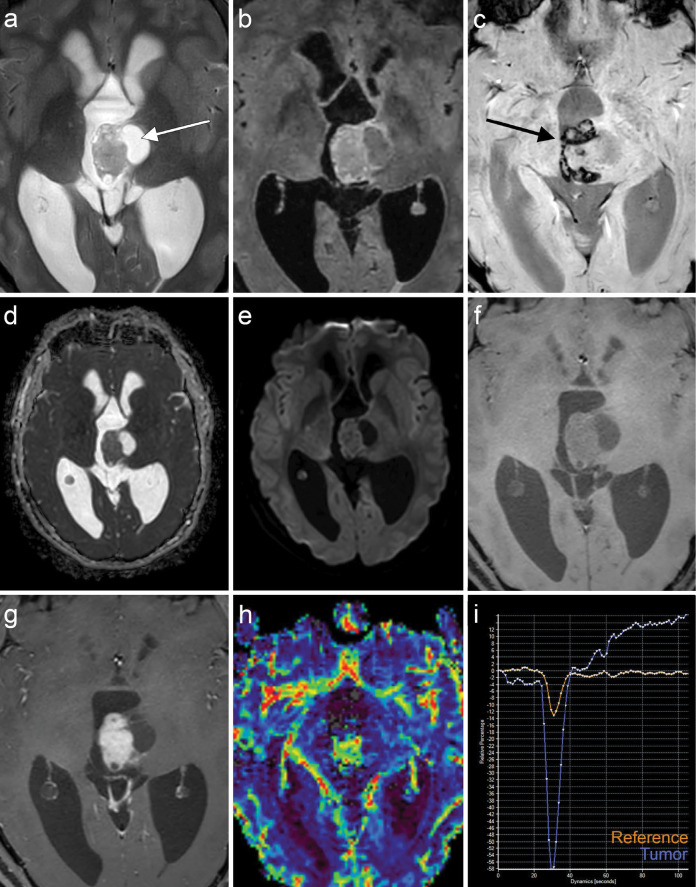
Magnetic resonance images (MRI) of the *EWSR1::PATZ1* gene fusion tumor in the third ventricle. **(a)** T2 weighted images (T2‑WI) and **(b)** fluid attenuated inversion recovery (FLAIR) images show the multicystic mass, with incomplete suppression of the content of the cysts (arrow) on T2‑FLAIR. **(c)** Susceptibility‑weighted imaging (SWI) shows central and peripheral signal loss caused by calcifications and hemosiderin deposition (arrow). Apparent diffusion coefficient maps **(d)** and b1000 diffusion‑weighted images **(e)** show that the tumor exhibited facilitated diffusion compared with the gray matter with T2 shine through. T1‑WI before **(f)** and after **(g)** gadolinium contrast injection reveals intense contrast enhancement. Dynamic susceptibility contrast perfusion map **(h)** and curve **(i)** confirm relative hyperperfusion of the tumor (blue line) compared with normal white matter (orange line) with contrast leakage.

A computed tomography (CT) scan was performed to rule out intracranial hypertension or hemorrhage. The CT scan revealed a mass in the third ventricle, causing hydrocephalus by obstructing the cerebral aqueduct. The tumor appeared slightly hyperdense compared with normal white matter and contained multiple cystic components with coarse central calcifications. Magnetic resonance imaging (MRI; Figure 1) showed that the mass was hyperintense on T2‑weighted images (Figure 1a) and had a multicystic appearance with cysts of varying sizes and incomplete suppression of cyst contents on T2‑weighted fluid‑attenuated inversion recovery (FLAIR) images (Figure 1b). Susceptibility‑weighted imaging (Figure 1c) with phase maps revealed signal loss at the periphery of the mass, likely due to hemosiderin deposition, as well as central signal loss due to calcifications. The tumor demonstrated facilitated diffusion compared with gray matter, with T2 shine‑through and an apparent diffusion coefficient of 1.05 x 10^‑3^ mm²/s (Figure 1d–e). On T1‑weighted imaging, the tumor was hypointense compared with white matter and exhibited avid enhancement following gadolinium administration (Figure 1f–g). Dynamic susceptibility contrast perfusion imaging showed characteristics of a hyperperfused lesion (Figure 1h), with a significantly elevated relative cerebral blood volume corrected for contrast leakage, measuring 7.5 times that of normal white matter (Figure 1i). The post‑bolus portion of the curve continued above the baseline, indicative of contrast leakage.

**Figure 2:**
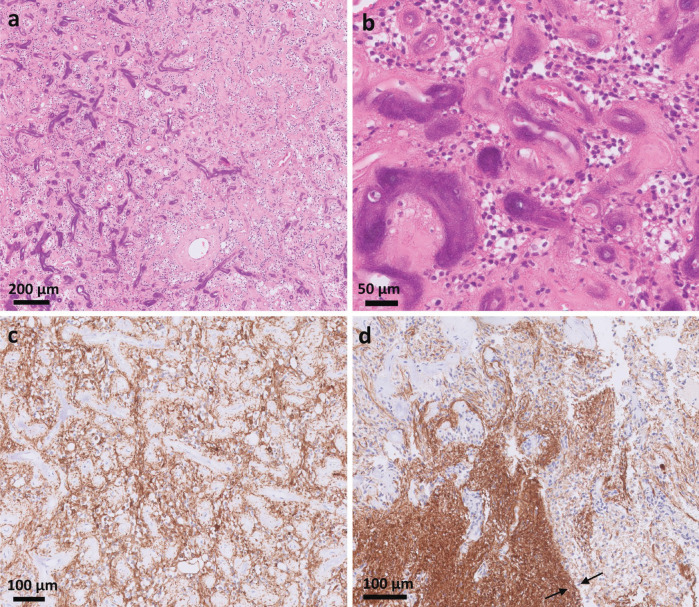
Histological slides of the tumor. Hematoxylin and eosin (H&E) stain overview image **(a)** shows numerous hyalinized blood vessels with partial calcification, mainly on the left side of the image (stained dark). A more magnified H&E stain **(b)** exhibits the hyalinized and partially calcified blood vessels with only limited tumoral cells between them. Glial fibrillary acidic protein (GFAP) stain **(c** and **d)** shows the positive tumor cells and the border of the lesion with the surrounding brain tissue (between the arrows).

A ventriculoperitoneal drain was inserted, and an endoscopic biopsy was performed. The biopsy was complicated by a hemorrhage. Histopathological examination (Figure 2) revealed hypercellular glial tissue with extensive hemorrhagic material and multiple calcifications. The increase in cellularity resulted from atypical cells with enlarged hyperchromatic nuclei, without notable mitotic activity or necrosis. The atypical cells infiltrated the surrounding preexisting glial tissue, and the lesion displayed extensive vascularization, including multiple, occasionally calcified, hyalinized capillaries interspersed between the cells. The histopathological diagnosis was a glial tumoral process with low‑grade appearance whose nature and biological behavior were unclear. Further RNA‑based sequencing (FusionPlex Expanded Sarcoma kit, ArcherDx) revealed the presence of an *EWSR1::PATZ1* fusion. IDH mutation analysis was negative.

Because there is no consensus regarding the correct treatment of this type of tumor, treatment was started with temozolomide and radiotherapy on the basis of the article by Ene et al. [2]. A total of 1 year after treatment, there was a significant reduction in the solid enhancing components and a slight enlargement of the cystic components.

## Methods

We reviewed the literature to find common imaging characteristics using the query “*EWSR* AND *PATZ*” on PubMed. Inclusion criteria were (1) an explicit diagnosis of an *EWSR1::PATZ1* gene fusion CNS tumor, (2) MRI images or a description of imaging findings, and (3) publication in the English language.

## Results

**Figure 3:**
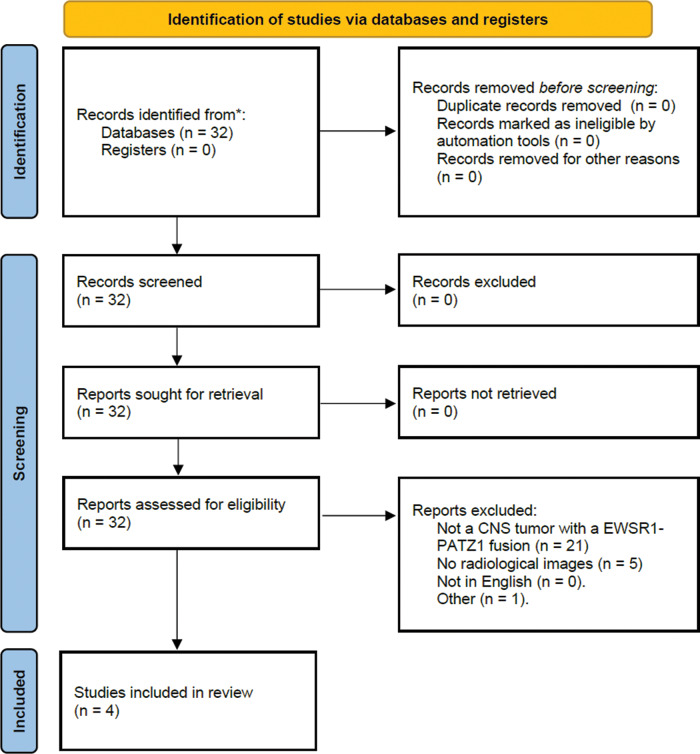
PRISMA flowchart.

The Preferred Reporting Items for Systematic Reviews and Meta‑analyses (PRISMA) flowchart is provided in Figure 3. One article technically met the inclusion criteria but was excluded because it described a tumor that developed the *EWSR1::PATZ1* fusion many years later, at the recurrence as a glioblastoma (GBM), making it unlikely that the fusion was the genetic driver mutation [2]. Ultimately, 4 of the 32 articles were included, yielding a total of six cases. All articles featured contrast‑enhanced T1‑weighted images and three out of four articles contained a T2‑weighted sequence. None of the articles contained pre‑contrast T1‑weighted images, diffusion‑weighted images, susceptibility‑weighted images, or perfusion curves. The common imaging characteristics are summarized in Table 1.

**Table 1:** Imaging features of the *EWSR1::PATZ1* gene fusion CNS tumors included in this study.

AGE (YEARS) AND SEX	DX (HISTOLOGY)	LOCATION	T2	C+	CA^++^	SOLID/CYSTIC	DELINEATION	OTHER	REFERENCE
24, m	Glial tumor	Third ventricle	+	++	+	Mixed	Circumscribed	Hydrocephalus	Current study
32, f	Papillary glioneuronal tumor (low‑grade)	Left lateral ventricle	+	++	Microscopic on pathology	Mixed	Circumscribed	Hydrocephalus	[5]
7, f	Ganglioglioma (low‑grade)	Fourth ventricle	+	++	Microscopic on pathology	Mixed	Circumscribed	Hydrocephalus	[5]
13, f	Astroblastoma	Right lateral ventricle	+	++	+	Mixed	Circumscribed	Hydrocephalus; novel *MN1::GTSE1* gene fusion	[9]
37, f	Ependymoma (anaplastic) WHO 3	Cerebellar cortical		++		Mixed	Circumscribed	Mild hydrocephalus	[6]
57, m	Ependymoma WHO 2	Third ventricle		++		Mixed on path.	Circumscribed	Hydrocephalus	[6]
1.4, m	Infantile glioblastoma	Right lateral ventricle	+	++	Microscopic on pathology	Mixed	Circumscribed	Hydrocephalus right ventricle; leptomeningeal involvement	[7]

m = male; f = female; C+ = Contrast enhancing; CA++ = calcifications; MN1::GTSE1 gene fusion = Meningioma 1 ‑ G2 and S‑Phase Expressed 1 gene fusion.

## Discussion

*EWSR1::PATZ1* gene fusion central nervous system (CNS) tumors are a potential novel entity, with only 21 cases reported and an as‑yet‑unknown prevalence [2, 4–9]. The imaging characteristics of these tumors are not well established owing to their rarity. In this study, we present a detailed description of the imaging characteristics of *EWSR1::PATZ1* fusion CNS tumors and compare them with cases reported in the literature to identify common imaging patterns, as described in Table 1.

The four primary common imaging patterns include (1) intense contrast enhancement; (2) small and large intratumoral cysts; (3) intraventricular location or extension, often associated with hydrocephalus; and (4) sharp delineation. Additionally, internal calcifications or hemorrhagic foci were frequently observed.

Firstly, intense contrast enhancement was consistently observed in every reviewed case, indicative of significant disruption of the blood–brain barrier. High intratumoral relative cerebral blood volume (rCBV) values are likely a result of pronounced vascular proliferation often seen in histological examinations [4–8]. This neoangiogenesis may also contribute to the multiple hemorrhagic foci detected on pathological examination and imaging. Secondly, The small and large intratumoral cysts in our case are believed to be a consequence of small intratumoral hemorrhages, but they could also result from cystic degeneration or necrosis. Thirdly, all but one of the evaluated tumors were located in or exhibited extension into the ventricles. The exception was a tumor centered at the cerebellar cortex, which caused hydrocephalus due to compression of the fourth ventricle and was histopathologically diagnosed as an ependymoma [6]. While our tumor displayed an infiltrative pattern on histology, it appeared well circumscribed on imaging. This was a common feature in all included tumors and was pathologically confirmed in one case [5]. Lastly, macroscopic calcifications were observed in two out of seven cases, while microscopic calcifications were noted in three additional cases on histology. The presence of calcifications was not mentioned in the two cases diagnosed as ependymomas [6].

Tumors harboring the *EWSR1::PATZ1* fusion are predominantly observed among children, adolescents, or young adults, with a median age of 20.4 years [2]. However, there is a wide age spectrum, with the youngest patient being 17 months old and the oldest being 57 years old [6, 7].

The oncogenic mechanism of the *EWSR1::PATZ1* fusion remains unclear. The *PATZ1* gene is involved in maintaining embryonic stem cells in an undifferentiated state by inhibiting neural differentiation and regulating cellular reprogramming [3, 4]. The *EWSR1* gene is prone to breakage and translocation and has various fusion partners. While *EWSR1* itself is not described as oncogenic, it causes its fusion partners to be overexpressed [5, 6, 8]. There is some evidence that increased *PATZ1* expression may confer resistance to temozolomide, potentially carrying important therapeutic implications [2, 10]. Compared with classic glioblastomas, *EWSR1::PATZ1* tumors generally exhibit better survival, although data remain limited owing to their rarity [2, 4, 8].

The primary limitation of this study is its small sample size, primarily owing to the rarity of *EWSR1::PATZ1* fusion tumors, resulting in limited available data from case studies. The heterogeneous histological appearance further complicates the classification of these tumors as a distinct tumor type or as a versatile anomaly capable of manifesting in various types of CNS tumors [1]. While additional published data are needed, the increasing utilization of molecular diagnostics may lead to an increase in the prevalence of these tumors in the future.

In conclusion, *EWSR1::PATZ1* gene fusion tumors exhibit diverse histopathological appearances, yet certain consistent imaging features can be identified. These features include avid contrast enhancement, sometimes accompanied by intralesional hemorrhagic foci, the presence of cysts within the lesions, intraventricular extension resulting in hydrocephalus, and the occurrence of calcifications. If these tumors indeed represent a distinct entity, recognizing these common features can be instrumental in the workup of these tumors, as radiologists can be the first to propose the diagnosis.
